# Activation of Magnesium Lignosulfonate and Kraft Lignin: Influence on the Properties of Phenolic Resin-Based Composites for Potential Applications in Abrasive Materials

**DOI:** 10.3390/ijms18061224

**Published:** 2017-06-08

**Authors:** Lukasz Klapiszewski, Artur Jamrozik, Beata Strzemiecka, Danuta Matykiewicz, Adam Voelkel, Teofil Jesionowski

**Affiliations:** 1 Poznan University of Technology, Faculty of Chemical Technology, Institute of Chemical Technology and Engineering, Berdychowo 4, PL-60965 Poznan, Poland; artur.robert.jamrozik@gmail.com (A.J.); beata.strzemiecka@put.poznan.pl (B.S.); adam.voelkel@put.poznan.pl (A.V.); teofil.jesionowski@put.poznan.pl (T.J.); 2 Wielkopolska Centre of Advanced Technologies, Umultowska 89 C, PL-61614 Poznan, Poland; 3 Poznan University of Technology, Institute of Material Technology, Division of Plastic Processing, Piotrowo 3, PL-61138 Poznan, Poland; danuta.matykiewicz@put.poznan.pl

**Keywords:** magnesium lignosulfonate, kraft lignin, activation agents, abrasive tool components, physicochemical and morphological characteristics

## Abstract

Magnesium lignosulfonate and kraft lignin were activated by different oxidizing agents for use in phenolic resin composites used for the production of abrasive components. The physicochemical properties of the oxidized materials were analyzed by Fourier transform infrared spectroscopy (FTIR), X-ray photoelectron spectroscopy (XPS), dynamic mechanical-thermal analysis (DMTA) and inverse gas chromatography (IGC). The homogeneity of the model abrasive composites containing the studied products was assessed based on observations obtained using a scanning electron microscope (SEM). FTIR and XPS analysis of the oxidized products indicated that the activation process leads mainly to the formation of carbonyl groups. The IGC technique was used to assess changes in the surface energy and the acid–base properties of the studied biopolymers. The changes in the acid–base properties suggest that more groups acting as electron donors appear on the oxidized surface of the materials. DMTA studies showed that the model composites with 5% magnesium lignosulfonate oxidized by H_2_O_2_ had the best thermomechanical properties. Based on the results it was possible to propose a hypothetical mechanism of the oxidation of the natural polymers. The use of such oxidized products may improve the thermomechanical properties of abrasive articles.

## 1. Introduction

Lignin was one of the first biopolymers to be discovered. The first mention of lignin as an “encrusting material” in wood comes from Payen in 1839. About 20 years later, the term “lignin” came to refer to the material known today [1]. Nowadays, lignin is considered mostly as a waste or by-product of the wood pulping process [2]. Every year 50 million tons out of a potential 300 million tons of lignin is extracted, but only about 2% is used for further applications, the rest being burnt for energy [3,4]. It should be noted, however, that because of its aromatic structure, lignin appears to be an abundant, sustainable and renewable source of raw materials for added-value chemicals [5].

The exact structure of native lignin has not been fully determined, but its basic building blocks are well known. Lignin is composed of three types of phenylpropanoid (C_6_–C_3_) units—*p*-coumaryl alcohol, coniferyl alcohol and sinapyl alcohol [6,7].

There are many types of commercial lignins, and all of them differ significantly from native lignin. Most of the lignin on the market comes from the popular kraft alkali process for paper pulp production [8]. A second significant process, in which lignin is obtained in the form of lignosulfonates, is the sulfite pulping method [1,9].

The reactivity of lignin depends largely on the functional groups present in its structure. The most common groups in lignin are methoxyl groups, phenolic and aliphatic hydroxyl groups and aldehyde groups. Unfortunately, the vast majority of the functional groups are occupied in interunit linkages. There are several methods that can be used for activation or modification of these groups. Modification can be carried out by acidolysis [10,11], acetylation [12,13], alkylation [14,15], halogenation [16,17], nitration [18,19], sulfonation [20,21] and ozonolysis [22,23]. Another way of increasing lignin’s reactivity is the selective oxidation of β-*O*-4 linkages using nitrobenzene [24,25,26], potassium permanganate [27,28], sodium periodate [29,30] or hydrogen peroxide [31,32].

Phenolic hydroxyl groups, in particular, are occupied in interunit linkages such as carbon-oxygen bonds (β-*O*-4, α-*O*-4, 4-*O*-5). The greatest influence on the reactivity of lignin comes from chemically unstable ether bonds between β carbon and 4 carbon, which represent 35–70% of all linkages present in the biopolymer macromolecule [33]. Breaking a β-*O*-4 bond can be a relatively uncomplicated way to improve lignin’s reactivity [34,35]. Oxidation with potassium permanganate, sodium periodate or hydrogen peroxide may be helpful in achieving the desired effect. This kind of modification makes it possible to unlock occupied functional groups (mostly methoxyl groups and phenolic hydroxyl groups) capable of reacting with other polymers. Polymers that can easily react with unoccupied functional groups in lignin include phenolic resins—novolacs and resoles. Lignin can be incorporated into the structure of phenolics and strongly bind them, similarly to the bonding of cellulose fibers in wood.

Phenolic resins offer excellent properties and comprehensive binding capabilities with various substances. They are suitable for many applications. Currently, the most common use for these resins is as adhesives for bonding various structural wood products [36]. They are also used as binders in the manufacture of bonded abrasives [37]. However, phenolic resins also have disadvantages. During annealing and thermal curing volatile organic compounds (VOC), which are highly dangerous to human life, can be emitted [38]. With the development of technology and increasing environmental awareness, scientists are looking for solutions that improve the performance of manufactured products while limiting their impact on the environment. Lignin, due to its affinity to phenols, appears to be an interesting material for phenol replacement in phenolic resins. Such materials are increasingly used for research and in industrial processing, in order to lower production costs and reduce toxicity.

Phenol is one of the most widely used raw materials in the chemical industry, with annual production of approximately 8.7 million tons in 2008, and 97% of it is produced using the cumene process [37]. In recent years a significant increase in exploitation of bio-based phenol from lignin degradation can be observed, which can be beneficial for both industry and the environment. In some cases, phenol has been replaced with hardwood lignins from bamboo and eucalyptus in plywood manufacturing [39]. Such samples had a slightly lower shear strength than reference samples without lignin, but they had lower rates of wood failures. It is also possible to replace almost 50% of the phenol in phenol formaldehyde (PF) resins with softwood kraft lignin when preparing oriented strand boards, without any decrease in mechanical properties [40]. Nevertheless, kraft lignin is not the only type of lignin that may be used as a phenol replacement in PF resins. Lignins from sulfite pulping, which are mostly salts of Ca, Mg, Na and NH_4_, can be used in the preparation of wood panel adhesives, making it possible to reduce the pressing time in comparison with commercially used PF resins [41]. The use of lignosulfonates can be beneficial even in such demanding applications as brake pads. PF resins modified with 40% lignosulfonates exhibited better wear results and a higher friction coefficient [42]. Unfortunately, there are some serious problems with applying plant-derived oligomeric/polymeric lignin derivatives on a mass scale. In many cases, attempts to replace fossil-derived phenols can be unsuccessful due to the low reactivity of unmodified lignin with formaldehyde due to low phenolic hydroxyl content and linked to steric hindrance preventing formaldehyde attack [43]. Current efforts are focused on chemical transformation of lignin to increase the ratio of active hydroxyl groups, or cross-linking both kraft lignin and organosolv lignins with crosslinking agents other than formaldehyde, such as furfuryl alcohol or glutaraldehyde [44].

The main aim of this work is the modification and comprehensive description of ecological, functional fillers based on renewable kraft lignin and magnesium lignosulfonate, and determination of the thermomechanical properties of model abrasive composites incorporating the prepared oxidized lignins. It can be expected that the application of such preparations in phenolic-based composites may: (i) limit the emission of free phenol from phenolic resins composites through partial replacement of phenolic resins via oxidized lignin in composites used for abrasive tool production as well as limit the emission of formaldehyde (the oxidized lignin as active filler may react with formaldehyde present in phenolic resins); and (ii) improve the viscoelastic properties of the resulting composites. Activated lignin may be a very promising environmentally friendly and renewable material for sustainably developing the modern abrasives industry.

## 2. Results and Discussion

### 2.1. Fourier Transform Infrared Spectroscopy

**Figure 1:**
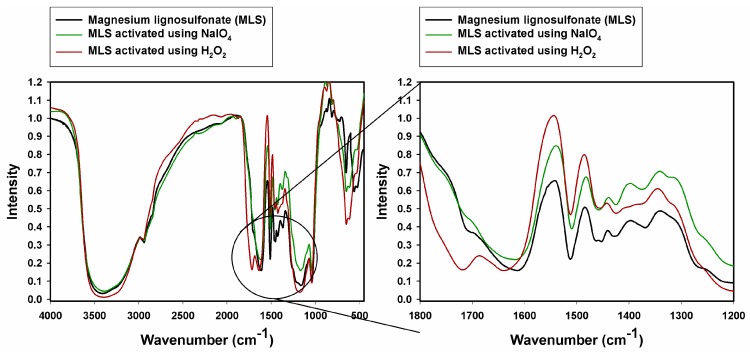
FTIR spectra of pure and activated magnesium lignosulfonate.

**Figure 2:**
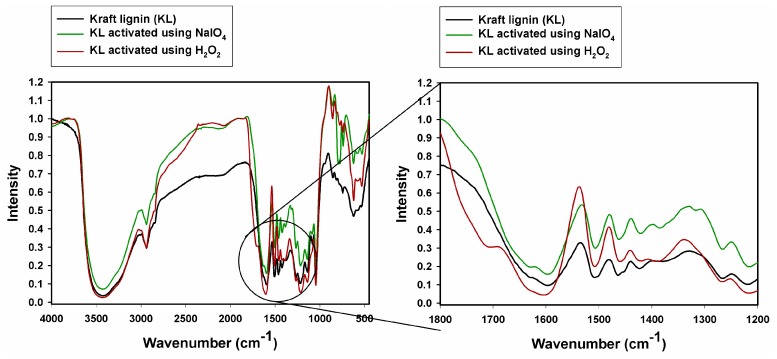
FTIR spectra of pure and activated kraft lignin.

Fourier transform infrared spectroscopy (FTIR) was used to detect the functional groups of magnesium lignosulfonate and kraft lignin, both non-modified and oxidized. Full spectra for lignosulfonate and lignin are presented in Figure 1 and Figure 2, respectively. Characteristic wavenumbers and corresponding vibration types of the tested compounds are given in Table 1.

For all lignin materials, characteristic bands were detected: stretching vibrations of aromatic hydroxyl groups at 3450–3380 cm^−1^, C–H_x_ stretching vibrations from methyl and methylene groups at 2950–2930 cm^−1^ and vibrations derived from aromatic rings in the lignin structure at 1620–1600 cm^−1^, 1518–1509 cm^−1^ and 1430–1400 cm^−1^. The observed values are in accordance with literature data [45,46]. Most of the bands are characteristic for the phenylpropanoid units present in the lignin structure, especially the most common guaiacyl unit [47].

It should be expected that the oxidation of lignin would lead mainly to the formation of carbonyl groups (a band at wavenumber ~1700 cm^−1^). The carbonyl group band was observed only in the case of oxidation by H_2_O_2_, both for lignosulfonate and kraft lignin. Similar results were reported in [48] for rice straw lignin and [49] for sugarcane bagasse lignin, where oxidation using H_2_O_2_ caused a significant increase in the intensity of bands corresponding to unconjugated ketone and carboxyl group stretching vibrations at about 1710 cm^−1^. For magnesium lignosulfonate the effect is more explicit than in the case of kraft lignin, as can be clearly seen in Figure 1 and Figure 2. Carbonyl groups can also be expected after the oxidation of lignin by NaIO_4_.

### 2.2. X-ray Photoelectron Spectroscopy

**Figure 3:**
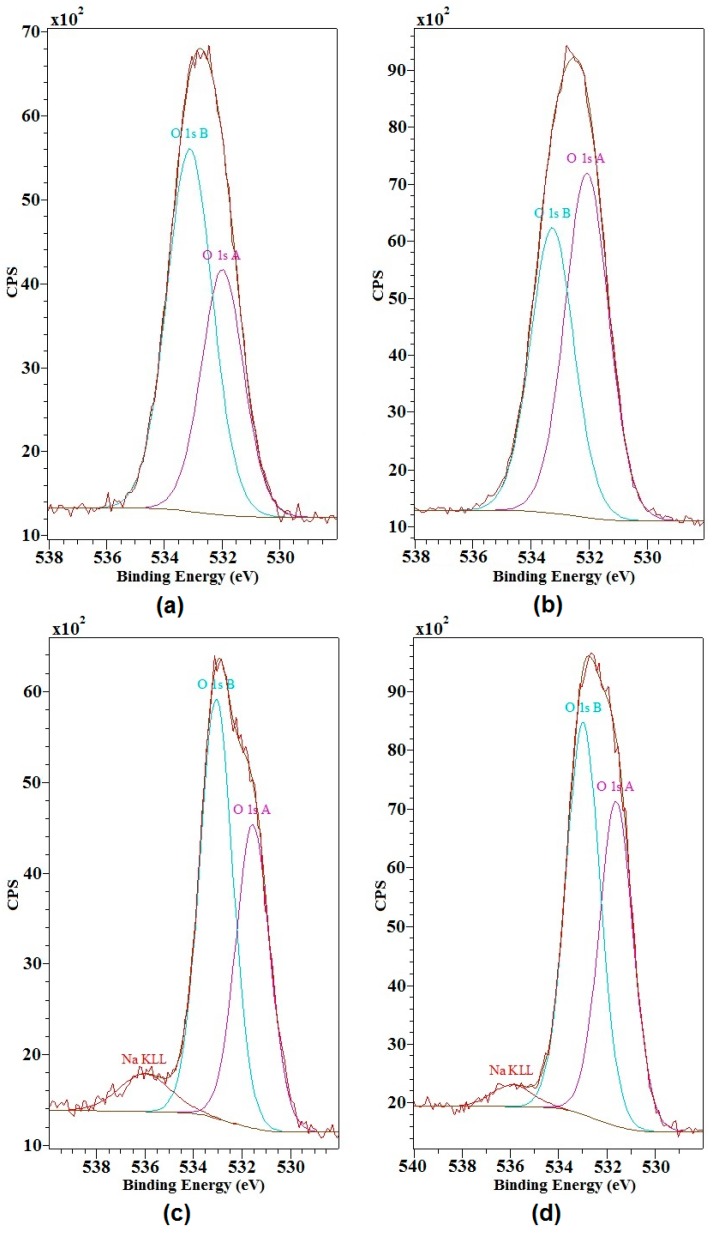
Spectra of the O 1s XPS region for: (**a**) magnesium lignosulfonate (MLS); (**b**) MLS activated using H_2_O_2_; (**c**) kraft lignin (KL); and (**d**) KL activated using H_2_O_2_.

The surface composition for all samples was examined by means of X-ray photoelectron spectroscopy. The surface of all samples is composed of carbon and oxygen, and, in the case of lignosulfonate samples, also sulfur. The elemental compositions are given in Table 2. Sulfur was also detected, as well as traces of sodium and magnesium, but these are not considered in the quantitative calculations. All samples exhibit peaks centered at ~285 and ~533 eV assigned to C 1s and O 1s respectively. Besides carbon and oxygen, kraft lignin was contaminated with sulfur and sodium, which may be a result of its preparation via the kraft process, as was observed in [50]. The evaluation of oxidation efficiency is based on analysis of the XPS O 1s peak. The spectra obtained have relatively complex profiles (see Figure 3).

Deconvolution of the experimental data was performed using a model consisting of four basic components of the C 1s transition: C 1s A–C 1s D. Component C 1s A, with a binding energy of 284.7 eV, corresponds to non-functionalized carbon atoms from aromatic rings present in the lignin structure. Component C 1s B, with binding energy 286.2 ± 0.2 eV, can be assigned to a set of groups with a carbon atom bonded to one atom of oxygen e.g., C–O–C, C–OH. Component C 1s C, not present in kraft lignin when unmodified or activated using NaIO_4_, shifted by 1.1 ± 0.1 eV from component C 1s B in the direction of increasing binding energies, is attributed to carbon atoms from carbonyl groups. Component C 1s D, present in all samples, with binding energy 288.9 ± 1.3 eV, is attributed to carbon atoms bonded in O=C–O– functional groups. The results are in accordance with those previously reported by us [51,52]. The atomic concentration of carbon located in carbonyl groups in the case of magnesium lignosulfonate is higher for both activated samples by 1.1–1.5%. For kraft lignin when unmodified or activated using NaIO_4_, the signal from carbonyl groups is not present; however, the signal is visible for the sample oxidized using H_2_O_2_, which confirms that this oxidation process was successful.

The most valuable evidence of the success of oxidation can be observed in the XPS O 1s spectra. Example survey spectra and O 1s regions of both unmodified magnesium lignosulfonate and MLS activated using H_2_O_2_ are shown in Figure 3. Component O 1s A, with binding energy 532.0 ± 0.3 eV, corresponds to oxygen atoms from carbonyl groups in the magnesium lignosulfonate samples. The kraft lignin samples had the component O 1s A shifted by 0.5 ± 0.3 eV in the direction of decreasing energy. Component O 1s B, with binding energy 533.0 ± 0.3 eV, corresponds to carbon atoms from C–O groups. In all cases activation caused an increase in the atomic concentration of oxygen atoms from carbonyl groups. The largest increases were observed for samples activated using H_2_O_2_ (70% for magnesium lignosulfonate, 50% for kraft lignin).

The results of XPS analysis can be considered as confirmation of the formation of new carbonyl groups in the structure of magnesium lignosulfonate and kraft lignin. It can be concluded that the activation process was performed successfully.

### 2.3. Hypothetical Mechanisms of Activation of Lignin and Lignosulfonate

**Figure 4:**
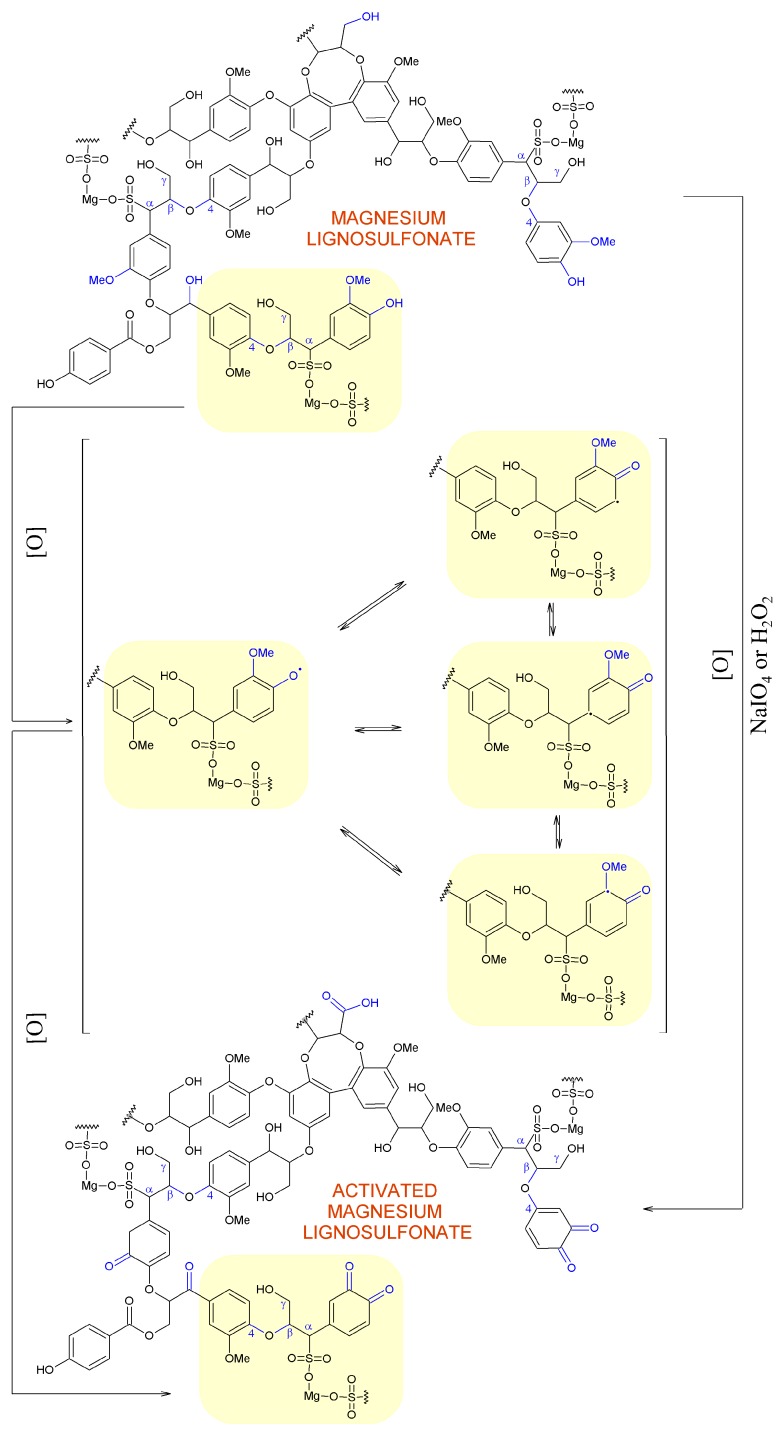
Proposed mechanism of activation of magnesium lignosulfonate using NaIO_4_ and hydrogen peroxide.

**Figure 5:**
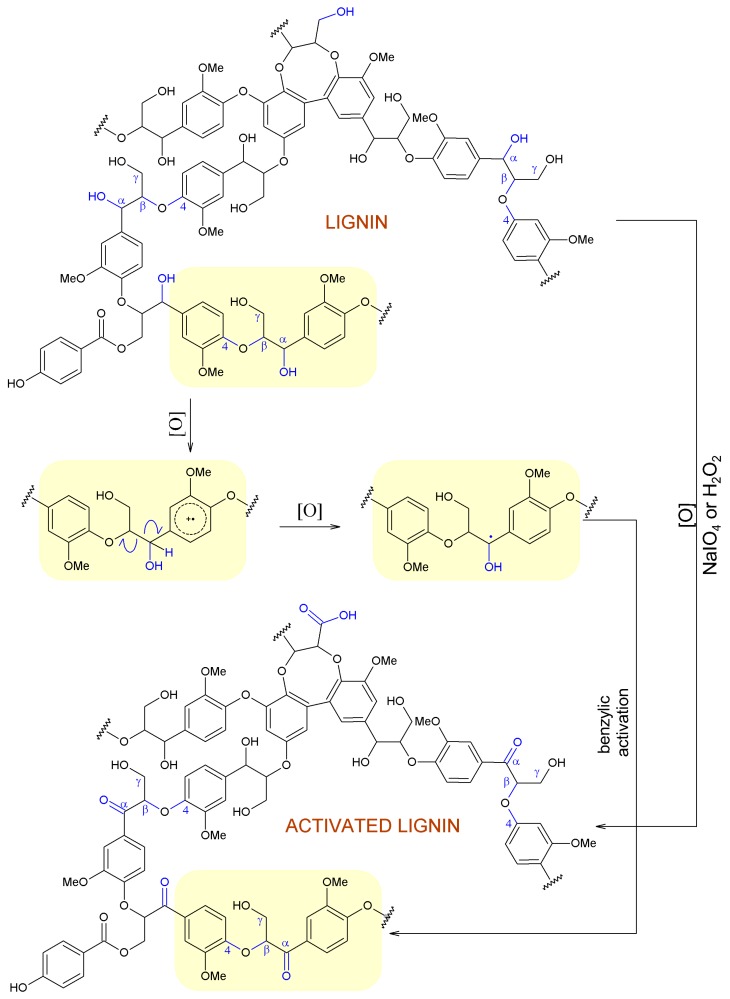
Proposed mechanism of activation of kraft lignin using NaIO_4_ and H_2_O_2_.

Based on the results obtained by Fourier transform infrared spectroscopy and X-ray photoelectron spectroscopy as well as literature data [1], hypothetical mechanisms of the activation of magnesium lignosulfonate and kraft lignin using NaIO_4_ and H_2_O_2_ can be proposed. The proposed mechanism for activation of magnesium lignosulfonate is shown in Figure 4, and that for kraft lignin in Figure 5. As a result of the modification of lignin using NaIO_4_ and H_2_O_2_ the hydroxyl groups present at the C-α, especially for β-O-4 bonds, may be activated [1]. Moreover, it may be suspected that –CH_2_OH groups connected to aromatic rings may be transformed into –C=O or –COOH. Transition states in the mechanism of interactions are presented in Figure 5. A similar mechanism of interaction, taking into account the transition states, is presented for magnesium lignosulfonate. In this case, the oxidation produces methoxy and hydroxyl groups on the aromatic ring, as shown in Figure 4, and quinone is formed.

### 2.4. Dynamic Mechanical-Thermal Properties

Determination of the mechanical behavior of composites containing lignin preparations with increasing temperature was performed using dynamic mechanical-thermal analysis (DMTA). The values of the composite storage modulus (G’) at various temperatures, highest loss factor (tan δ_max_) and glass transition temperature (T_g_) are given in Table 3. The highest value of G’ (3.45 GPa) was recorded for the composite with MLS activated using H_2_O_2_. The significant increase in the storage modulus of the composite with MLS activated using H_2_O_2_ may be attributed to the generation of a strong interface between the hydroxyl groups of the lignin activated using H_2_O_2_ and the phenolic hydroxyl groups of the polymer matrix [53]. The lowest value of G’ (1.15 GPa) was recorded for the composite with KL activated using NaIO_4_.

For all investigated composites, with the exception of the composite with MLS, the glass transition temperature was approximately 250 °C. Despite the fact that for all samples the mechanical properties began to decline at a temperature of about 150 °C, the composite with KL activated using NaIO_4_ exhibited the highest glass transition temperature, at approximately 254 °C, compared with 235 °C for the composite with MLS. Lignin can act as a thermoplastic as well as a thermosetting material. At elevated temperatures, lignin creates cross-linked structures and behaves as a thermosetting material [54,55].

The highest loss factor for all samples is most visible in the temperature range 150–250 °C. The damping properties described by tan δ correspond to the molecular motions existing in the materials [56]. DMTA method gives possibility to detect all changes in the state of molecular motion in polymer composites in a defined temperature range [57]. The thermal transitions in polymers can be characterized as free volume changes or relaxation times. Amorphous materials such as resins transform from a glassy state to rubbery state with the temperature rise [58].

**Figure 6:**
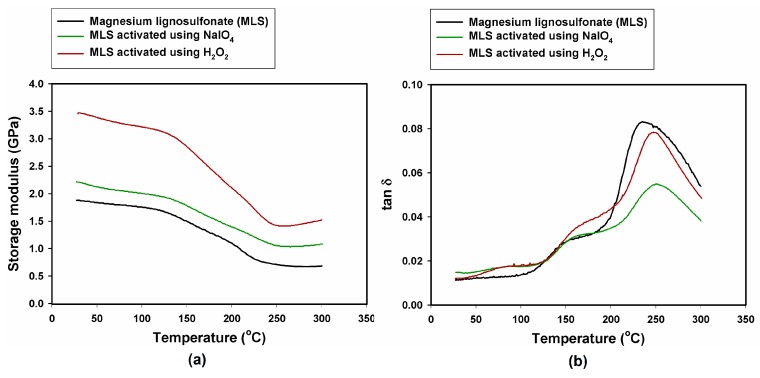
Mechanical-thermal properties of composites with lignosulfonates: (**a**) storage modulus; and (**b**) loss factor.

It can be inferred that this process begins at a temperature of 150 °C, when the storage modulus starts to decrease, and ends at approximately 250 °C, when the sample is vitrified. At this temperature, the loss factor is the highest for all samples. The highest width of the tan δ curve was observed for the composite with MLS activated with H_2_O_2_, which may demonstrate the improved ability of the composite to dissipate energy through molecular motion [59]. The storage modulus and tan δ curves of the composites with lignosulfonate are shown in Figure 6.

**Figure 7:**
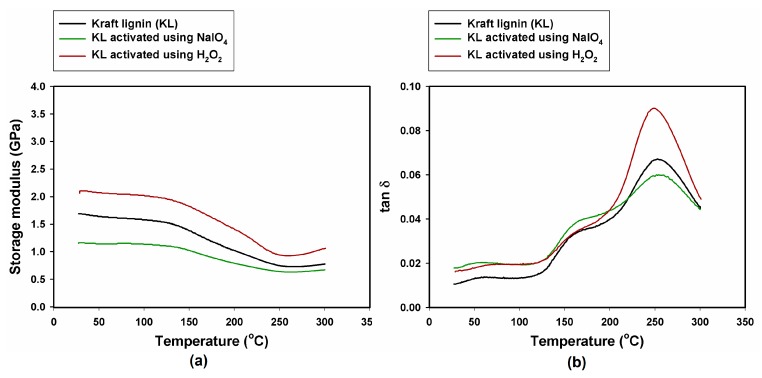
Mechanical-thermal properties of composites with kraft lignin: (**a**) storage modulus; and (**b**) loss factor.

All of the composites with kraft lignin exhibited lower storage modulus values than the samples with lignosulfonate. Figure 7 shows the G’ and tan δ curves obtained for composites with the addition of kraft lignin.

The lowest value of G’ is that of the sample with KL activated using NaIO_4_. For all samples, as in the case of lignosulfonate, mechanical properties began to decline at a temperature of approximately 150 °C. The decrease is most marked for the sample containing KL activated with H_2_O_2_. This sample also has the highest loss factor. The glass transition process begins at 150 °C and ends at around 250 °C. It can be noted that phenolic resin in combination with lignosulfonate gives better results than with kraft lignin. In addition, available bibliographic information indicates that the thermomechanical properties of phenol resins depend on the curing conditions and the structures of the hardeners and fillers [60,61].

### 2.5. Inverse Gas Chromatography

**Figure 8:**
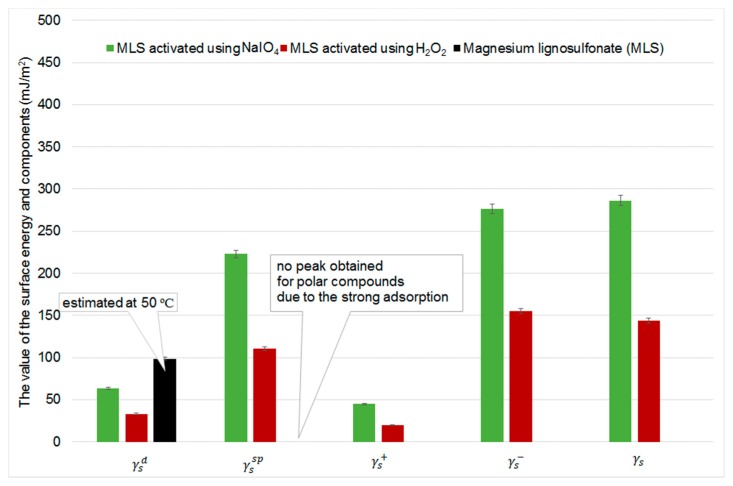
Surface free energy and its components for lignosulfonate samples.

The oxidation process has a significant influence on the surface properties under all of the studied conditions. It affects lignosulfonate and kraft lignin differently. Lignosulfonate was characterized by a very active surface: adsorption at 30 °C was so strong that no peaks were obtained for alkanes from octane to nonane and for all polar compounds used. The surface properties of lignosulfonate were therefore tested at 50 °C, but polar compounds were still very strongly adsorbed. Such strong interactions with polar test compounds in the case of lignosulfonate may be the result of ionic groups Mg^2+^ −SO_3_–R(Ar). After oxidation, the surface energy decreases in all cases, which may be a result of hydrolysis of sulfonate groups. This effect is dominant, and the effect of oxidation of –R–OH groups is not observed as an increase in the specific component of surface energy. Values of surface free energy components for the lignosulfonate samples are presented in Figure 8.

**Figure 9:**
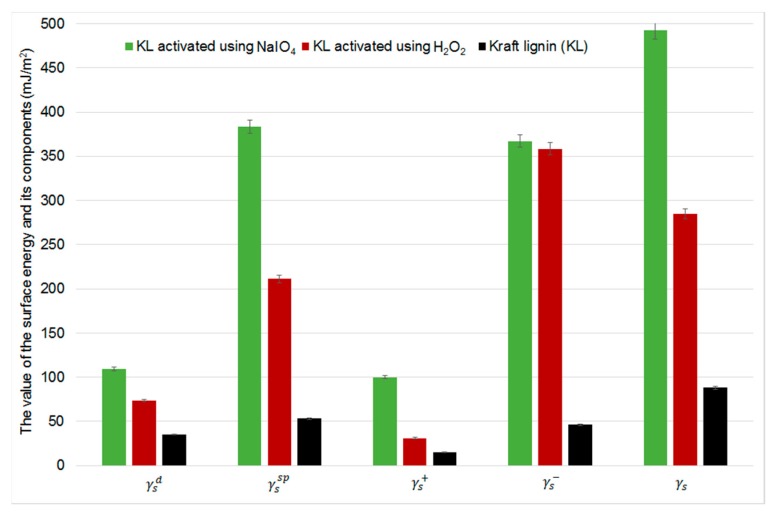
Surface free energy and its components for kraft lignin samples.

Oxidation increases kraft lignin’s surface energy, especially the specific component $\left(\gamma_{s}^{\mathrm{sp}}\right)$. The largest increase in this component is observed for KL activated using NaIO_4_. Surface energy parameters obtained for lignin samples are presented in Figure 9.

In the case of kraft lignin, –CH_2_OH groups are oxidized to –COOH groups, and this is reflected in an increase in the ability of the surface to donate electrons (an increase in $\gamma_{s}^{-}$), and also to act as an acid (the H in the carboxyl group is more acidic than in the hydroxyl group). Considering the changes in the surface properties determined by IGC, NaIO_4_ was found to be a weaker oxidizing agent. The value of the $\gamma_{s}^{d}$ parameter measured for kraft lignin (35.2 mJ/m^2^) is in agreement with the values reported in the literature [62]: from 30 to 39 mJ/m^2^ at 40 °C depending on the kraft pulp. Belgacem et al. reported slightly higher values of $\gamma_{s}^{d}$ for kraft lignin (46 mJ/m^2^) but that material came from a different source than that used in the present study [63]. This suggests that kraft lignin has different surface properties depending on its source. In [63], it is also reported that the value of $\gamma_{s}^{d}$ for lignosulfonate is much higher than for other lignins (67 mJ/m^2^ at 50 °C), but lower than the value of the $\gamma_{s}^{d}$ parameter reported here (98 mJ/m^2^ at 50 °C; this concerns a different type of lignosulfonate, however). An increase in $\gamma_{s}^{d}$ after oxidation of the lignocellulosic fibers by sonication was also reported in [64]; the authors explained it by the higher activity of the oxidized lignocellulose surface.

### 2.6. Scanning Electron Microscopy

**Figure 10:**
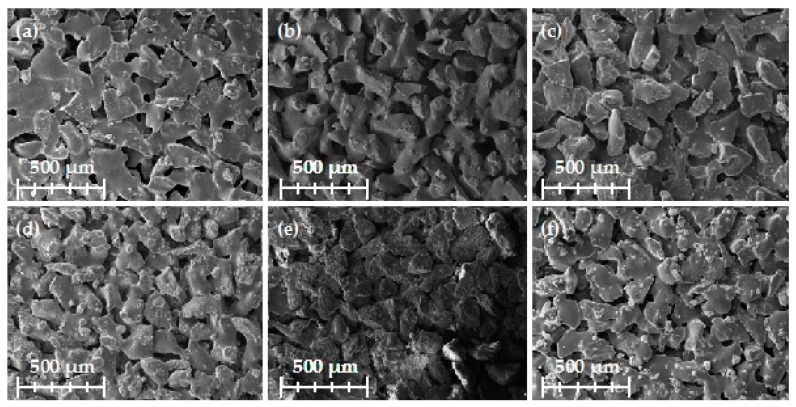
Scanning electron microscopy images of examined composites: (**a**) MLS; (**b**) MLS activated using NaIO_4_; (**c**) MLS activated using H_2_O_2_; (**d**) KL; (**e**) KL activated using NaIO_4_; and (**f**) KL activated using H_2_O_2_.

A scanning electron microscope (SEM) was used to examine the surface morphology of the composites following DMTA analysis. The SEM images showed all of the composites to have a uniform structure (Figure 10). In all cases the lignin materials were located at the interface between the grain surface and organic resin; this is necessary for the proper performance of their function. All prepared composites had a porous structure, which is beneficial in the case of grinding composites. Pores provide access for coolants and lubricants, and increase grinding efficiency and tool life.

**Figure 11:**
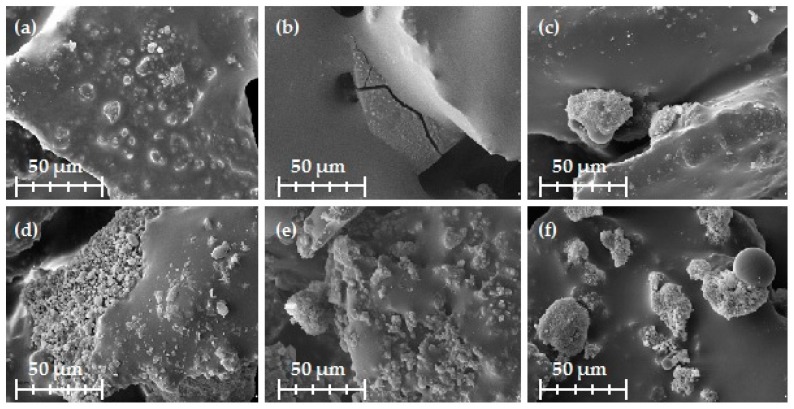
Scanning electron microscopy images of examined composites: (**a**) MLS; (**b**) MLS activated using NaIO_4_; (**c**) MLS activated using H_2_O_2_; (**d**) KL; (**e**) KL activated using NaIO_4_; and (**f**) KL activated using H_2_O_2_.

In samples with both pure and activated kraft lignin the filler exhibited a tendency to agglomerate, which can be clearly seen in Figure 11d–f. This may be caused by the stronger cohesion forces and the weaker adhesion forces to phenolic resins in the case of lignin macromolecules, which have a lower oxidation state, compared with lignosulfonates, which are more susceptible to oxidation. In the case of MLS samples, this phenomenon was not observed.

It must be noted that the samples with MLS activated using NaIO_4_ had undesirable surface imperfections such as micro-cracks which were not present in the other samples. This can impair the mechanical parameters because of the damage to the aromatic structure of the phenylpropanoid units in lignosulfonate, but the precise effect on the properties is not fully known and is currently the subject of our intensive research. In the case of kraft lignin, this phenomenon did not occur.

## 3. Materials and Methods 

### 3.1. Modification of Kraft Lignin and Magnesium Lignosulfonate

In the study kraft lignin (Sigma-Aldrich, Steinheim am Albuch, Germany) and magnesium lignosulfonate (Vianplast 55, Biotech Lignosulfonate Handels Gmbh, St. Valentin, Austria) were used. Each biopolymer was modified using sodium periodate (NaIO_4_) and hydrogen peroxide (H_2_O_2_) in a quantity of 7.5% by mass. Oxidation was carried out as follows: 5 g of lignin or lignosulfonate material was dissolved in 50 cm^3^ of water and stirred for 10 min, then 30 cm^3^ of oxidant solution (2.5% concentration) was added to the reaction flask using a peristaltic pump. After 1 h, the pH of the mixture was lowered to about 4 using sulfuric acid, as is done in the lignin precipitation process [65,66,67]. This step made it possible to precipitate lignin and filtrate it from the mixture containing oxidants. After this step the lignin was washed with acidified (H_2_SO_4_, pH approximately 4) water and double distilled water to remove residual sulfuric acid. Finally, the filter cake was transferred to a vessel and placed in a convection dryer with forced air circulation (Memmert, Schwabach, Germany) for 24 h. The resulting dried powder was triturated in a mechanical mortar and sieved.

### 3.2. Preparation of Composites

The tested composites consisted of 80% electrocorundum grains (95A) with a granulation of 120 mesh (Imerys, Mannheim, Germany), 12% novolac (with 9% hexamine) binder (Lerg S.A., Pustków, Poland), 5% activated lignin material, and 3% resole (Lerg S.A., Pustków, Poland). When the binder was changed the quantity and type of grains and wetting agent remained unchanged. The prepared mixtures were placed in PTFE molds, pressed between two steel plates. The molds were additionally bolted to prevent deformation. The prepared mold was then placed in a furnace, where it was annealed according to a specific temperature program: heating from 50 °C up to 180 °C, heating rate 0.2 °C/min, then heating at 180 °C for 10 h.

### 3.3. Material Analysis

#### 3.3.1. Fourier Transform Infrared Spectroscopy

Fourier transform infrared spectroscopy (FTIR) was used to confirm the presence of new functional groups after the oxidation process. The apparatus used was a Vertex 70 spectrophotometer (Bruker, Karlsruhe, Germany). Analyzed samples had the form of tablets formed by placing a mixture of anhydrous KBr (250 mg) and 2 mg of the tested material between two steel rings under a pressure of 10 MPa. Measurements were performed at a resolution of 0.5 cm^−1^ in the wavenumber range 4000–400 cm^−1^.

#### 3.3.2. X-ray Photoelectron Spectroscopy

X-ray photoelectron spectra (XPS) were obtained using Al *K*α monochromatized radiation (h*υ* = 1486.6 eV) with a Prevac system (Rogów, Poland) equipped with a Scienta R4000 electron energy analyzer (Uppsala, Sweden) operating at constant transmission energy (Ep = 50 eV). The samples were loosely placed in a molybdenum sample holder. The analysis chamber during the experiments was evacuated to better than 1 × 10^−9^ mbar. Data processing involved background subtraction by means of an S-type integral profile and a curve-fitting procedure (a mixed Gaussian–Lorentzian function was employed) based on a least-squares method (CasaXPS software). The experimental errors were estimated to be ±0.2 eV for the photoelectron peaks of carbon and iron. Charging effects were corrected using the C 1s component ascribed after deconvolution to the aliphatic carbon bindings (Comp (2)) and set to 285.0 eV. The reproducibility of the peak positions thus obtained was ±0.2 eV.

#### 3.3.3. Dynamic Mechanical-Thermal Properties

The viscoelastic properties of the composites, described by the storage modulus (G′) and glass transition temperature (T_g_), were analyzed using dynamic mechanical-thermal analysis (DMTA) in torsion mode (Anton Paar MCR 301 apparatus, Graz, Austria). Samples with dimensions of 10 × 4 × 50 mm were investigated with the apparatus operating at frequency *f* = 1 Hz, over a temperature range from 25 to 300 °C with a heating rate of 2 °C/min. The position of tan δ at its maximum was taken as the glass transition temperature.

#### 3.3.4. Inverse Gas Chromatography

Measurements were carried out using an IGC surface energy analyzer (SEA) produced by SMS Ltd. (Glasgow, UK), equipped with a flame ionization detector. The carrier gas was helium, with a flow rate of 15 cm^3^/min. Glass-packed, silanized columns with 4 mm I.D. and 300 mm length were used in the experiments. The temperature of the detector and injector was 150 °C. All columns were conditioned overnight at the standard flow rate at 30 °C. Vapours of the test compounds were injected in quantities which ensured infinite dilution.

##### Determination of Dispersive Component of the Surface Free Energy

The dispersive component of the surface free energy ($\gamma_{S}^{D}$) was determined using n-alkanes as test compounds. The $\gamma_{S}^{D}$ value was determined by the Schultz–Lavielle method [68].

##### Determination of Specific Component of Surface Free Energy

The Good–van Oss equation is used to calculate the acidic ($\gamma_{S}^{+}$) and basic ($\gamma_{S}^{-}$) parameters of a solid surface. It is expressed by Equation (1):

(1) $$\Delta G^{sp}=2\mathrm{Na}+\sqrt{\gamma_{S}^{-}\cdot \gamma_{L}^{+}}$$

The symbols $\gamma_{L}^{-}$ and $\gamma_{L}^{+}$ denote the electron donor and electron acceptor of the test compound, and $\gamma_{S}^{-}$ and $\gamma_{S}^{+}$ denote the electron donor and electron acceptor of the studied material. Dichloromethane (DM) and ethyl acetate (EA) can be used as test compounds [69]. The acidic and basic character of the surface are calculated from Equations (2) and (3), respectively:

(2) $$\gamma_{S}^{-}={\left(\frac{\Delta G_{DM}}{2\cdot N_{A}\cdot a_{DM}}\right)}^{2}\cdot \left(\frac{1}{\gamma_{DM}^{+}}\right)$$

(3) $$\gamma_{S}^{+}={\left(\frac{\Delta G_{EA}}{2\cdot N_{A}\cdot a_{EA}}\right)}^{2}\cdot \left(\frac{1}{\gamma_{EA}^{-}}\right)$$

The values of $\gamma_{DM}^{+}$, $\gamma_{DM}^{-}$, $\gamma_{EA}^{+}$ and $\gamma_{EA}^{-}$ used for calculation are given in Table 4.

Knowing the values $\gamma_{s}^{+},\;\gamma_{s}^{-}$, the parameter $\gamma_{s}^{\mathrm{sp}}$, denoting a specific interaction as bipolar, H-bond type, metallic, acid–base or hydrophobic, is calculated according to:

(4) $$\gamma_{s}^{sp}=2\cdot \sqrt{\gamma_{s}^{+}\cdot \gamma_{s}^{-}}$$

The total surface energy $\left(\gamma_{s}\right)$ is the sum of the dispersive $\left(\gamma_{s}^{d}\right)$ and specific $\left(\gamma_{s}^{sp}\right)$ components.

#### 3.3.5. Scanning Electron Microscopy

The surface morphology and microstructure of the composites after DMTA studies (see description of composites in Section 3.2) were examined on the basis of SEM images recorded from an EVO40 scanning electron microscope (Zeiss, Oberkochen, Germany) at an accelerating voltage of 10,000 V, working distance 9900 μm and emission current 13,300 nA. Before testing, the samples were coated with Au for a time of 5 s using a Balzers PV205P coater (Oerlikon Balzers Coating SA, Brügg, Switzerland).

## 4. Conclusions

A relatively simple procedure for the oxidation of kraft lignin and magnesium lignosulfonate has been presented, and has been shown to be an effective method to obtain activated forms of the biopolymers. The most effective of the investigated oxidizing agents was H_2_O_2_. Based on XPS and FTIR analysis, it was possible to propose a hypothetical mechanism for the activation of kraft lignin and magnesium lignosulfonate by NaIO_4_ and H_2_O_2_. Lignosulfonate oxidized to quinone groups, whereas in kraft lignin hydroxyl groups present at the C-α are transformed to carbonyl groups. Magnesium lignosulfonate has high surface activity, which decreased after oxidation, probably due to the hydrolysis of sulfonate groups. Oxidation of kraft lignin causes an increase in the surface energy, but the changes are smaller than in the case of lignosulfonate. The main change in the surface properties when lignin was oxidized was an increase in its basicity. This may result from the formation of carbonyl groups during the oxidation process. These surface changes are reflected in the macroscopic behavior of the lignins in phenolic resin composites. Both kraft lignin and magnesium lignosulfonate agglomerated in the phenolic resin matrix. The model composite with lignosulfonate oxidized with the strong oxidant NaIO_4_ has microvoids visible on SEM images, which may be the result of damage to the aromatic structure of phenylpropanoid units in lignosulfonate, causing a decrease in the molecular strength of the polymer chains. The most homogenous composites are those with lignosulfonate or kraft lignin oxidized by H_2_O_2_. This may be one of the reasons why these composites exhibited the best thermo-mechanical properties (the highest value of G’). Thus, lignin oxidized with nontoxic hydrogen peroxide may be a promising material for use as a filler in abrasive articles.

## Figures and Tables

**Table 1:** Vibrational wavenumbers (cm^−1^) attributed to magnesium lignosulfonate (MLS), kraft lignin (KL), and biopolymers activated using NaIO_4_ and H_2_O_2_.

MLS	MLS NaIO_4_	MLS H_2_O_2_	KL	KL NaIO_4_	KL H_2_O_2_	Vibrational Assignment
3425	3390	3400	3426	3424	3447	O–H stretching
2940	2945	2940	2940	2942	2947	C–H_x_ stretching
-	-	1725	-	-	1705	C=O stretching
1610	1617	-	1600	1603	1612	C–C, C=C (aromatic skeleton), stretching
1515	1513	1514	1509	1517	1512	
1465	1460	1430	1465	1454	1465	C–H (CH_3_ + CH_2_), bending
1427	1430	-	1421	1427	-	C–C, C=C (aromatic skeleton), stretching
1379	1379	-		-	1396	
-	-	-	1271	1270	1272	C–O (guaiacyl unit) stretching
-	-	-	1219	1221	1224	C–OH (phenolic OH) stretching
1179	1170	1172	1143	-	1145	Aromatic C–H (guaiacyl unit), stretching
1044	1045	1035	1045	1050	1048	C–OH + C–O–C (aliphatic OH + ether) stretching, sulfonic acids
-	-	-	856	-	861	Aromatic C–H (guaiacyl unit), bending
-	-	-		777	-	Aromatic C–H (guaiacyl unit), bending
-	-	-	744	743	-	
654	649	662	-	627	624	CH_x_ bending

**Table 2:** Elemental composition of samples examined using XPS analysis.

Sample	Region	Name	Binding Energy (eV)	Atomic Concentration (%)	Bonds Assignment	Mass Concentration (%)
Magnesium lignosulfonate (MLS)	C 1s	C 1s A	284.7	65.4	C–C	63.0
		C 1s B	286.0	25.4	C–O	
		C 1s C	287.0	6.1	C=O	
		C 1s D	288.9	3.0	O=C–O–	
	O 1s	O 1s A	532.0	38.5	C=O	26.0
		O 1s B	533.1	61.5	C–O	
MLS activated using NaIO_4_	C 1s	C 1s A	284.7	50.1	C–C	47.4
		C 1s B	286.1	30.3	C–O	
		C 1s C	287.2	7.6	C=O	
		C 1s D	289.0	12.1	O=C–O–	
	O 1s	O 1s A	532.1	54.7	C=O	38.9
		O 1s B	533.3	45.3	C–O	
MLS activated using H_2_O_2_	C 1s	C 1s A	284.7	59.3	C–C	45.4
		C 1s B	286.0	28.8	C–O	
		C 1s C	287.1	7.2	C=O	
		C 1s D	288.9	4.7	O=C–O–	
	O 1s	O 1s A	532.3	65.6	C=O	29.4
		O 1s B	533.3	34.4	C–O	
Kraft lignin (KL)	C 1s	C 1s A	284.7	60.1	C–C	56.3
		C 1s B	286.3	35.7	C–O	
		C 1s C	288.2	4.2	O=C–O–	
	O 1s	O 1s A	531.5	42.6	C=O	30.7
		O 1s B	533.1	57.4	C–O	
KL activated using NaIO_4_	C 1s	C 1s A	284.7	50.9	C–C	55.8
		C 1s B	286.2	40.2	C–O	
		C 1s C	288.6	8.9	O=C–O–	
	O 1s	O 1s A	531.6	45.3	C=O	33.0
		O 1s B	533.0	54.7	C–O	
KL activated using H_2_O_2_	C 1s	C 1s A	284.7	54.5	C–C	39.3
		C 1s B	286.2	34.9	C–O	
		C 1s C	287.4	3.5	C=O	
		C 1s D	288.6	7.1	O=C–O–	
	O 1s	O 1s A	531.7	63.7	C=O	31.3
		O 1s B	533.1	36.3	C-O	

**Table 3:** Values of storage modulus (G’), highest loss factor (tan δ_max_) and glass transition temperature (T_g_) for the examined composites.

Sample, Model Abrasive Composite With:	G’ 25 °C (GPa)	G’ 50 °C (GPa)	G’ 300 °C (GPa)	tan δ_max_ (−)	T_g_ (°C)
MLS	1.88	1.84	0.68	0.083	235
MLS activated with NaIO_4_	2.22	2.12	1.08	0.055	250
MLS activated with H_2_O_2_	3.45	3.39	1.52	0.079	247
KL	1.69	1.64	0.78	0.067	252
KL activated with NaIO_4_	1.15	1.14	0.67	0.060	254
KL activated with H_2_O_2_	2.10	2.07	1.06	0.090	250

**Table 4:** Physicochemical data for the test compounds used; L means liquid as DM and EA.

Compound	Dispersive Component $\gamma_{L}^{D}\;\left(\frac{\mathrm{mJ}}{m^{2}}\right)$	Acidic Component $\gamma_{L}^{-}\;\left(\frac{\mathrm{mJ}}{m^{2}}\right)$	Basic Component $\gamma_{L}^{+}\;\left(\frac{\mathrm{mJ}}{m^{2}}\right)$
Dichloromethane	24.5	5.2	0.0
Ethyl acetate	23.9	0.0	6.2
